# Indorsement of Licenses from Other State Examining Boards

**Published:** 1895-05

**Authors:** 


### State Medical Examinations.

### INDORSEMENT OF LICENSES FROM OTHER STATE EXAMINING BOARDS.

UNIVERSITY OF THE STATE OF NEW YORK,
REGENTS' OFFICE, ALBANY.

MEMORANDUM.

THE laws, 1893, ch. 661, p. 148, provide that "applicants examined and licensed by other state examining boards registered by the regents as maintaining standards not lower than those provided by the article, . . . may without further examination, on payment of $10 to the regents and on submitting such evidence as they may require, receive from them an indorsement of their licenses."

*New Jersey*—The medical act provided May 22, 1894, requires in point of preliminary education "a competent common school education." This is interpreted as follows in the printed regulations of the state board of medical examiners: "Candidates must be graduated from an accredited literary or scientific college, or have completed satisfactorily not less than a three years' course in an accredited high school or academy or have received a preparatory education covering the following branches—namely, orthography, arithmetic, English grammar and composition, geography, history of the United States, algebra and physics, as certified by the state superintendent of public instruction of New Jersey." New Jersey, therefore, seems to require as a minimum only eight academic counts or two-thirds of one year in a high school, while New York requires fifty counts or three full academic years, except for those who actually pass regents' examinations, and even in these cases, as in dentistry, the New York examination standard will doubtless soon be advanced to a full high school course.

The secretary of the New Jersey board writes that the superintendent of public instruction will actually examine all who have not satisfactorily completed an accredited three-year academic course, but neither the law nor the published regulations fix any such requirement.

Though four years' study of medicine is required as compared with three years in New York, (in New Jersey one year's hospital service, or at least five years' reputable practice of medicine may be accepted as equivalent to one year's study,) and though the New Jersey medical examinations include more distinct topics than are specified in the New York law, yet New Jersey requires only a total average in all subjects of 75 per cent., provided that in no topic the percentage falls below 50, while the New York law requires 75 in *each topic.* The New York state board of medical examiners, to which the question of the professional requirements in New Jersey was referred, reports that though the question papers are apparently more severe than in New York, yet the comparatively few failures indicate a much lower standard of rating answers. The New Jersey state board of medical examiners requires a fee of $50 for indorsement of licenses from other states, whereas our law fixes the fee at $10. Under these provisions a new York applicant desiring a license for both states would be forced to pay $75, or $40 more than would be the fee were he to take the New Jersey examination and then apply for a license in New York.

*Pennsylvania.*—The act approved May 18, 1893, (to take effect March 1, 1894,) provides in point of preliminary education as in New Jersey, a competent common school education. July 26, 1894, the secretary of the state board of medical examiners wrote that no maximum or minimum standard in point of preliminary education had been established, but that the state medical society would require students to appear before an examining committee of the respective county societies, who would examine them before they entered on the study of medicine. July 30, 1894, the secretary wrote that the standard had been fixed "at an average education as evidenced by examinations in geography, spelling, history, grammar, composition and arithmetic," and that the state board of medical examiners would require from the candidates for admission to the licensing examination a certificate of the medical examiners of the respective county medical societies or a diploma or a certificate from a college, academy or high school.

It will be seen that the Pennsylvania requirements are still lower and more indefinite than in New Jersey. The Pennsylvania law requires three years' study of medicine (after July 1, 1895, four years,) and three courses of lectures in a legally incorporated medical college, but the statute makes no exaction as regards the character of the medical school attended. The law is, in fact, modeled after the first New York law and has, therefore, the defects which were remedied in New York in 1893. The Pennsylvania medical examinations are not as comprehensive as those in

New Jersey, and require only a general average of not less than 75 per cent., and not 75 per cent. in *each topic* as in New York.

The great difficulty in other states is the lack of a central educational and administrative body to pass on preliminary requirements and to supervise and conduct the examinations.

It would not be at all just to New York students to indorse licenses from other states unless, as the statute requires, the standards of these states are fully as high in all respects as that maintained in New York. The New York statutes give the regents power in all these applications to require candidates to submit such evidence as may be demanded. Certainly no action should be taken toward the indorsement of licenses from any other state examining board till a committee from New York has actually assisted in conducting the professional examinations, and both preliminary and professional credentials and answer papers have been critically examined in New York. Even after a favorable report along these lines, probably no license should be indorsed from any other state examining board, without requiring that preliminary and professional credentials be submitted and also all answer papers written at the examination at which the candidate was licensed.

